# Überlegungen zur Lockerung des Lockdowns

**DOI:** 10.1007/s10273-020-2635-1

**Published:** 2020-04-22

**Authors:** Michael Hüther, Hubertus Bardt

**Affiliations:** Institut der deutschen Wirtschaft Köln e.V., Konrad-Adenauer-Ufer 21, 50668 Köln, Deutschland

**Keywords:** O40, I15, H12

## Abstract

Die durch das Coronavirus verursachte Pandemie stellt ein beachtliches gesundheitliches Risiko dar. Zur Bewältigung dieser Krise müssen in erheblichem Umfang wirtschaftliche Ressourcen für das Gesundheitssystem eingesetzt werden. Angesichts der gesundheitlichen Risiken sind die hierfür aufzubringenden Kosten gegenwärtig nicht relevant. Wenn der Lockdown endet, ist jedoch mit erheblichen Anpassungsproblemen und Stockungen auf dem Weg zu einer wieder friktionsfrei arbeits-, risiko- und wissensteiligen Volkswirtschaft zu rechnen.

## Abstract (en)

The fight against the coronavirus pandemic has led to an insulation of social and economic life and will have considerable economic consequences. Important areas of the industry and service sectors were partially or completely shutdown. A resumption of activity should happen as soon as possible, once the medical pre-conditions have been established and are met. This requires a clear exit strategy and following several steps to return to previous welfare and growth data levels. After securing survival during this crisis via various liquidity lines and bridging loans, the economy’s restart requires the relaunch of public infrastructure, especially of schools and kindergartens. To facilitate a coordinated and synchronised restart of complex industrial value chains, we need clear signals on a planned schedule. A tax policy driven departure signal and a demand side focused growth programme could make an important contribution to a new economic dynamic after the crisis.

Um die exponentielle Verbreitung der lebensbedrohlichen Coronavirus-Erkrankung zu verhindern, ist das Herunterfahren des wirtschaftlichen und gesellschaftlichen Lebens notwendig (vgl. Tabelle 1). Seit dem 16.3.2020 sind bundesweit Schulen und Kindergärten geschlossen, soziale Kontakte werden auf ein Minimum reduziert. Seit dem 23.3.2020 gelten zudem verschärfte Kontaktbeschränkungen. Teilweise als Folge dieser Maßnahmen, teilweise auf direkte staatliche Anordnung hat sich die wirtschaftliche Aktivität erheblich verändert und verringert: Ein hoher Anteil von Büroangestellten arbeitet von zuhause aus, der Bahnverkehr ist eingeschränkt, der Flugverkehr ist zum Erliegen gekommen. Zahlreiche Geschäfte sind geschlossen, die Gastronomie und personenbezogene Dienstleistungen weitestgehend reduziert, alle größeren Veranstaltungen abgesagt – mit entsprechenden Konsequenzen für die damit verbundenen Unternehmen. Grenzübergänge sind teilweise gesperrt, ganze Branchen – vor allem die Automobilindustrie hat hier eine Schlüsselrolle – haben ihre Produktion heruntergefahren. Land- und Bauwirtschaft drohen Beschränkungen durch fehlenden Zugang von Saisonkräften und Subunternehmen aus Mittel- und Osteuropa nach Deutschland. Unternehmen aller Art leiden zum einen unter angebotsseitigen Problemen – durch Behinderungen der Wertschöpfungskette fehlen notwendige Vorprodukte und durch Reiserestriktionen und Übertragungsrisiken fehlen Mitarbeiter – und zum anderen leiden sie unter fehlender Nachfrage, die Vertriebskanäle sind verstopft und Absatzmärkte abgeschottet. Die Finanz- und die Wirtschaftspolitik haben darauf umfangreich und zeitnah reagiert.

* Vollständig überarbeitete 2. Version, work in progress. Die Autoren danken für Feedback und Anregungen.

Die Kosten dieses Lockdowns belaufen sich auf mindestens 5 % der Wirtschaftsleistung, solange dieser mit seiner produktionseinschränkenden Wirkung nur bis Ende April 2020 geht und die Belastung infolge gestörter Lieferketten danach nicht länger als zwei Monate anhält. Je länger diese notwendigen Maßnahmen andauern, desto höher sind die damit verbundenen wirtschaftlichen Kosten. Ein Lockdown bis in den Sommer hinein wird zu zweistelligen Einbrüchen der Wirtschaftsleistung führen, dann zunehmend verbunden mit erheblichen psychosozialen Folgen, häuslichen Konflikten, individuellen Depressionen und gesundheitlichen Belastungen.

Zur Einordnung der gesamtwirtschaftlichen Szenarien ist eine Darstellung der vierteljährlichen Entwicklung des Bruttoinlandsprodukts (BIP) in längerer Frist hilfreich (vgl. Abbildung 1 und 2). In beiden Varianten wird eine starke Erholung im Jahresverlauf unterstellt. Das ist optimistisch, denn es unterstellt den friktionsfreien Kaltstart der deutschen Volkswirtschaft. Die Maßnahmen der Wirtschafts- und Finanzpolitik sind überwiegend als Überbrückungshilfen (Kurzarbeitergeld, Steuerstundungen, Kredite) ausgestaltet, unterstellen also einen zeitlich überschaubaren Einbruch.

**Prof. Dr. Michael Hüther** ist Direktor des Instituts der deutschen Wirtschaft (IW) in Köln und Hochschullehrer an der European Business School in Oestrich-Winkel.

**Dr. Hubertus Bardt** leitet den Bereich Wissenschaft am IW in Köln und ist Lehrbeauftragter an der Rheinischen Fachhochschule Köln und der Heinrich-Heine-Universität Düsseldorf.

Tabelle 1
**Maßnahmen und Indikatoren in der Corona-Krise**

| Kalenderwoche | Politikintervention im Inland | Grenzregimeveränderung (EU) | | Wirtschaftspolitik | Wirtschaftliche Indikatoren | | |
|---|---|---|---|---|---|---|---|
| | | Mobilität von Personen | Internationaler Warenverkehr | | ifo-Geschäftsklimaindex[1] | Einkaufsmanagerindex[2] | IW-Umfrage[3] |
| 11 (9. März) | Absage Großveranstaltungen | | | 1) Erleichterung Bezug Kurzarbeitergeld (rückwirkend ab 1.3.)<br>2) Liquiditätsstützung angekündigt | Februar:<br>Geschäftsklima: 96,0<br>Lage: 93,0<br>Erwartungen: 3,1 | 4. März:<br>Verarbeitendes Gewerbe: 48,0<br>Dienstleistungen: 52,5 | Gesamt: 37 %<br>Industrie: 42 %<br>Dienstleistungen: 35 % |
| 12 (16. März) | Bund-Länder-Vereinbarung: Schulschließungen | BE, BG, CH, CRO, CZ, DK, ES, EST, FI, I, LUX, LV, N, NL, P, PT, RO, S, SK | BG, (CZ), (I), (P), (SK), | | 19. März:<br>Geschäftsklima: 87,7<br>Lage: 93,8<br>Erwartungen: 2,0 | | Gesamt: 67 %<br>Industrie: 68 %<br>Dienstleistungen: 69 % |
| 13 (23. März) | Bundesweit umfangreiches Kontaktverbot: Einschränkung:<br>1) Versammlungsfreiheit<br>2) freie Berufsausübung<br>3) Gewerbefreiheit<br>4) Schulpflicht<br>5) Religionsfreiheit | A, GR, LIT | (A) | 1) Gesetz zur Errichtung eines Wirtschaftsstabilisierungsfonds<br>2) Infektionsschutzgesetzänderung<br>3) Sozialschutzpaket<br>4) Gesetz zur Abmilderung der Pandemiefolgen im Zivil-, Insolvenz-, Strafverfahrensrecht<br>5) COVID19-Krankenhausentlastungsgesetz<br>6) Corona-Soforthilfe für (Solo-)Selbständige/kleine und mittlere Unternehmen | 25. März:<br>Geschäftsklima: 86,1<br>Lage: 93,0<br>Erwartungen: 9,7<br>Verarbeitendes Gewerbe: -18.2<br>Dienstleistungen: -7,8<br>Handel: -21,4<br>Bau: 5,0 | 24. März:<br>Verarbeitendes Gewerbe: 45,7<br>Dienstleistungen: 34,5 | Gesamt: 59 %<br>Industrie: 63 %<br>Dienstleistungen: 63 % |

Anmerkung: A = Österreich, BE = Belgien, BG = Bulgarien, CH = Schweiz, CRO = Kroatien, CZ = Tschechien, DK = Dänemark, ES = Spanien, EST = Estland, FI = Finnland, GR = Griechenland, I = Italien, LIT = Litauen, LUX = Luxemburg, LV = Lettland, N = Norwegen, NL = Niederlande, P = Polen, PT = Portugal, RO = Rumänien, S = Schweden, SK = Slowakei; Länderkürzel in Klammern: eingeschränkte Regelungen.

[1] Ifo Konjunkturumfragen. [2] IHS Markit/BME Einkaufsmanager Index (EMI), 50 = keine Veränderung zum Vormonat. [3] Jeweils stark betroffene Unternehmen.

Quelle: eigene Zusammenstellung.

## Schwierigkeiten des Exits: Nichtlinearitäten, Rückkopplungen und Netzwerkeffekte

Unter der theoretischen Annahme, dass an einem Tag X alle Beschränkungen des gesellschaftlichen und wirtschaftlichen Lebens aufgehoben werden, ist mit erheblichen Anpassungsproblemen und Stockungen auf dem Weg zu einer wieder friktionsfrei arbeits-, risiko- und wissensteiligen Volkswirtschaft zu rechnen. Variabel bleibt nur die Dauer des Lockdowns. Komplexer wird die Reaktivierung der wirtschaftlichen Prozesse und Interaktionen, wenn – was sehr wahrscheinlich ist – die verfügten Eingriffe sukzessive aufgehoben werden. Die Vorstellung, dies als politische, gar sozialtechnologische Optimierungsaufgabe zu verstehen, dürfte sich schnell als Illusion erweisen. Eher ist davon auszugehen, dass die Aufhebung von Restriktionen sich stärker nach epidemiologischen Erwägungen richtet als nach ökonomischen Kriterien. Besonders aber die politischen Abstimmungsprozesse auf europäischer und internationaler Ebene dürften zusätzliche Friktionen begründen.

- Aus epidemiologischer Sicht wird aus Vorsichtsgründen nur ein schrittweises Aufheben der restriktiven Maßnahmen möglich sein.

- Die Krise trifft die verschiedenen Länder nicht gleichzeitig. Daher wird es noch über mehrere Monate Komplikationen in der Wertschöpfungskette geben, wenn in anderen Ländern Produktion ausfällt oder grenzüberschreitender Handel und Transport beschränkt sind.

Vor diesem Hintergrund kann nur skizziert werden, wie sich die Entwicklungen in der Krise in den volkswirtschaftlichen Sektoren vollziehen und welche qualitativen Problemverschärfungen drohen. Gesellschaftliche Kosten und individuelle Belastungen sind dabei nicht berücksichtigt. Der Unterschied zur oft zitierten Finanzkrise 2008/2009 liegt darin, dass die Ursachen diesmal zwar nicht im Ökonomischen liegen und Märkte durch Fehlsteuerung ihre Koordinationskraft verloren haben, aber wegen der Symmetrie des Pandemie-Schocks eine endo-

Abbildung 1

**Bruttoinlandsprodukt Deutschland – Szenario I: Q2 -10 %, im Jahresverlauf 2020 -5 % (2021 +4 %)**

preis-, saison-, arbeitstäglich bereinigt, 2015 = 100, in % gegenüber Vorjahr/Vorquartal

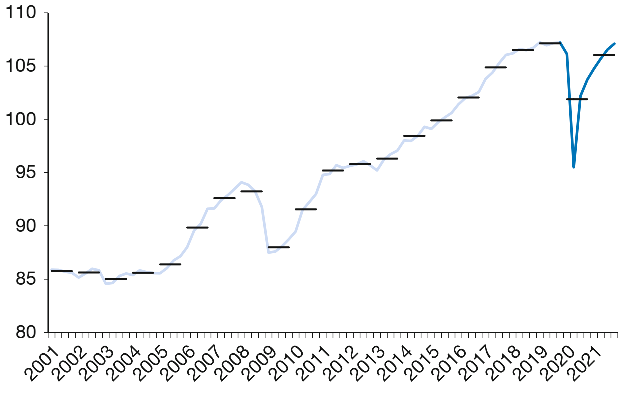

Quelle: eigene Berechnungen.

Abbildung 2

**Bruttoinlandsprodukt Deutschland – Szenario II: Q2 -20 %, im Jahresverlauf 2020 -10½ % (2021 +8 %)**

preis-, saison-, arbeitstäglich bereinigt, 2015 = 100, in % gegenüber Vorjahr/Vorquartal

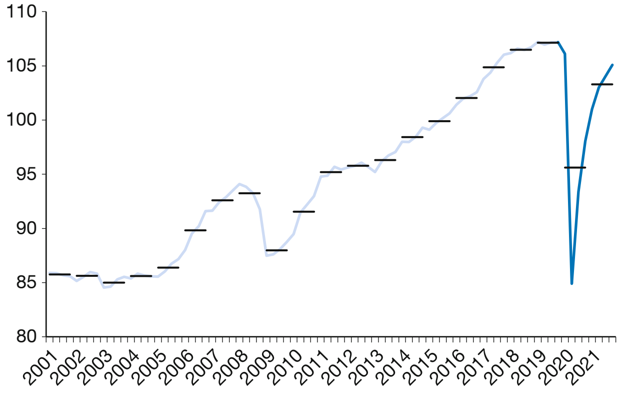

Quelle: eigene Berechnungen.

gene ökonomische Verschärfung mit zunehmender Dauer des Lockdowns droht:

- *Unternehmenssektor*: Mit unbestimmter Dauer des Lockdowns schreitet die Auszehrung der wirtschaftlichen Einheiten voran, zumal dann die einmaligen Zuschüsse des Bunds und der Länder zur Liquiditätsstärkung und Solvenzsicherung ihre Wirkung verlieren: Bei den Unternehmen wird zunächst das Eigenkapital angegriffen, für das Fremdkapital werden gegebenenfalls Überbrückungs-, Stundungs-, Umfinanzierungslösungen gefunden. Der Eigenkapitalverzehr mindert die wirtschaftliche Kraft des Unternehmens für eine künftige Expansion und verbaut/erschwert das Leveraging durch Fremdkapital. In die gleiche Richtung wirkt, dass die Überbrückungskredite über die Kreditanstalt für Wiederaufbau (KfW) und die Förderbanken der Länder zwar kurzfristig bestandssichernd sind, aber letztlich bei vielen Betrieben in einen Schuldenüberhang münden. Daran ändert auch die 100 %ige Besicherung durch den Steuerzahler nichts. Insgesamt dürfte die Unternehmensersparnis kurzfristig sinken, der Unternehmenssektor wird wieder zu einem Nettokreditnehmer – mittelfristig kann dies aber einen Deleveraging-Prozess in Gang bringen, der die Investitionen weiter schmälert. Es können zudem massive bilanzielle Bewertungsprobleme auftreten.

- *Private Haushalte*: Mit einem länger anhaltenden Lockdown werden die verfügbaren Einkommen der privaten Haushalte nachhaltig und nicht nur vorübergehend (abgefedert durch Kurzarbeitergeld) geschmälert. Es drohen Massenarbeitslosigkeit (2,5 Mio. bis 3 Mio.), Lohn- und Gehaltskürzungen, möglicherweise in hoher Zahl Privatinsolvenzen, was wieder den Bankensektor belasten würde. Die Nachfrage nach langlebigen Konsumgütern würde weiter massiv einbrechen, der Sozialkonsum und die personenbezogenen Dienstleistungen müssten mit anhaltend schwacher Nachfrage rechnen. Eine endogene Gesundung dieser besonders betroffenen Bereiche – soweit die Unternehmen überlebt haben – ist deshalb immer unwahrscheinlicher. Es kommt dann sehr darauf an, welche Maßnahmen der Konsumstützung ergriffen werden können (z. B. Gutscheine). All dies spricht für eine geringere Ersparnis, während die Bewertungsverluste an den Finanzmärkten zu einer höheren Ersparnis führen können, um die Absicherung im Alter zu gewährleisten. Der Nettoeffekt ist unklar.

- *Bankensystem*: Jede Umfinanzierung des Fremdkapitals von nichtfinanziellen Unternehmen schwächt das Bankensystem, weil Aktiva in der einen oder anderen Form an Wert verlieren werden. Die Verlagerung auf Förderbanken begründet die Haftung durch den Steuerzahler und befördert eine Re-Nationalisierung der Finanzintermediation. Die Zahl der Insolvenzen im Unternehmensbereich dürfte vor allem bei Kleingewerbetreibenden, Einzelunternehmern, Solo-Selbständigen, aber zunehmend auch bei den kleinen und mittleren Unternehmen steigen (zwar liegt hier die Eigenmittelquote mit rund 35 % deutlich höher als bei den ganz kleinen, doch wird diese irgendwann auch erodiert sein). Hinzu kommen die erwähnten Privatinsolvenzen und die Not vieler privater Haushalte, Ersparnisse aufzulösen und Bewertungsverluste zu realisieren. Kreditgeschäft und provisionsträchtiges Geschäft würde für die Banken auf längere Sicht einbrechen. Die Existenz von Regionalbanken dürfte ebenso kritisch werden wie von international tätigen Geschäftsbanken. Die für den Erholungsprozess notwendige Finanzintermediation dürfte kaum zu sichern sein; es sind umfassende nationale und internationale Restrukturierungen im Bankenmarkt zu erwarten.

- *Staatsfinanzen*: Da es sich um einen symmetrischen Schock handelt, sind alle Staaten gezwungen, mehr oder weniger parallel und zeitgleich zu handeln. Geht es zunächst um Stützungsmaßnahmen, so sind in einer späteren Phase erhebliche Rekapitalisierungen (Nichtbanken wie Banken) notwendig sowie umfangreiche Stützungsprogramme für den privaten Konsum (z.B. Halbierung der Mehrwertsteuer auf langlebige Konsumgüter) und die unternehmerischen Investitionen. Als notwendig kann sich auch erweisen, dass die krisenbedingten Schulden der Unternehmen übernommen werden (wie 1990 in einen Altschuldenfonds). Die Kommunen werden flächendeckend in Finanzierungsschwierigkeiten geraten, sodass erneut deren Handlungsfähigkeit gefährdet ist. Die Nettokreditaufnahme wird dramatisch ansteigen, sodass die Kapitalmärkte zu deutlichen Bonitätsdifferenzierungen gezwungen sein werden. Zur gesamtwirtschaftlichen Krise gesellt sich eine Staatsschuldenkrise, für deren Remedur die Notenbanken nicht mehr gut aufgestellt sind.

All dies führt zu der Einschätzung, dass ein V-Profil für die wirtschaftliche Erholung nur bei einem maximal bis Anfang Mai 2020 gehenden Stillstand plausibel ist. Ansonsten droht ein U-Verlauf mit langer Talsohle. Und diese kann von unterschiedlichen Dynamiken geprägt sein, wenn ab einem bestimmten Zeitpunkt eine Bankendestabilisierung unvermeidbar hinzukommt oder eine Staatsschuldenkrise droht und die Finanzierungskreisläufe in der Eurozone gefährdet sind. Mit solchen Rückkopplungen und Nichtlinearitäten haben wir keinerlei Erfahrung aus historischen Vergleichsfällen. So ist bei längerem Lockdown auf die selbstverstärkenden Kräfte zu achten, wenn beispielsweise die konjunkturelle (durch Nachfrageausfall bedingte) Arbeitslosigkeit durch Kapitalmangelarbeitslosigkeit verschärft wird, weil den Unternehmen der Finanzierungsspielraum für neue Anlagen und Ausrüstungen fehlt. Dieses Risiko steigt in dem Maße, in dem in Europa eine zeitliche Koordinierung der Exit-Phase nicht gelingt, um die Offenheit der Grenzen für den Warenverkehr (und die grenznahe Arbeitskräftemobilität) sicherzustellen.

Eine besondere Herausforderung liegt in der Orchestrierung des Neustarts, damit dieser nicht zum stotternden Kaltstart wird. Eine Aufgabe, die der Staat nur begleiten kann, aber die Koordinierung der Wirtschaft fordert. Positiv ist, dass der Kapitalstock anders als nach längerer Kriegswirtschaft oder einem Systemwechsel wie 1989/1990 in dieser Krise nicht obsolet geworden ist. Dessen Re-Aktivierung hängt wesentlich davon ab, welche fiskalische Kraft man dem Staat zutraut, um die Auslastung – ganz in der Argumentation von J. M. Keynes – glaubhaft sicherzustellen. Von Seiten des Kapitalangebots dürfte infolge weiter steigender Präferenz für sichere Anlagen (Vorsichtskasse) der geforderte Zins für die Zeichnung von Staatsanleihen sehr niedrig bleiben. Es scheint nicht unplausibel, dass die organisatorische Flexibilität, welche die Krise erfordert (Homeoffice, veränderte Meeting- und Kommunikationskultur), positive Folgen für die Produktivität hat. Die Digitalisierung führt über kapitalsparenden technischen Fortschritt endlich zu den erwarteten Produktivitätseffekten und begründet neue Wachstumshoffnung.

Zu beachten ist schließlich, dass das Ende des Lockdowns nicht alles wieder auf Normalzustand bringt, sondern es sicher durch weiter bestehende Einschränkungen gekennzeichnet sein wird. Umso wichtiger ist es, die Lockdown-Phase auf das medizinisch notwendige zu beschränken und zugleich eine klare Perspektive darüber zu geben, wie die weiteren politischen Schritte aussehen werden. Dort, wo noch keine zeitlichen Perspektiven aufgezeigt werden können, sollte zumindest deutlich gemacht werden, von welchen Indikatoren die weiteren Schritte abhängen. Dies steigert Vertrauen und unterstützt die wichtige Erwartungsbildung von Bürgern und Unternehmen. Deshalb erscheint es sinnvoll, für die verschiedenen Phasen vorzudenken:

- Eindämmungsphase: Lockdown,
- Öffnungsphase: sukzessiver Ausstieg aus den Eingriffen in die Grundrechte,
- Stabilisierungsphase: Suche nach dem „neuen Normal“,
- Wachstumsphase: Weg frei machen für Investitionen.

## Gestaltung der Öffnungsphase

Aktuell liegt die Herausforderung in der Gestaltung der Öffnungsphase, in der die Bestimmungen des Lockdowns schrittweise aufgehoben werden. Für eine wirtschaftspolitische Exit-Strategie aus dem Corona-Lockdown Ende April sind die epidemiologischen Voraussetzungen (Verflachung des Anstiegs der „Infizierten“, Testen auf das Virus, Identifizierung von Kontaktpersonen, Tracking/Tracing und Isolierung von Infizierten) als erfüllt anzusehen. Die Zahl der aktiven Fälle nimmt zwar immer noch zu, doch hat sich die Dynamik deutlich verlangsamt (vgl. Abbildung 3). Derzeit verdoppelt sich diese Zahl in Deutschland alle 17 Tage, aber auch in den anderen betrachten Staaten ist der Verlauf ähnlich.

Abbildung 3

**„Active Cases“: Infizierte abzüglich Genesener**

Veränderungen in % gegenüber Vortag, Stand: 13.4.2020

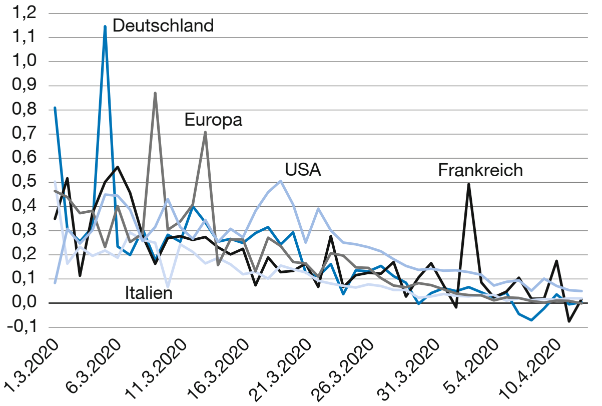

Quelle: Worldometer, https://www.worldometers.info/coronavirus (15. April 2020); eigene Berechnungen.

Der Einstieg in die Öffnung sollte so vollzogen werden, dass die einzelnen Maßnahmen von hoher gesellschaftlicher und wirtschaftlicher Wirksamkeit sind und epidemiologisch beherrschbar respektive kontrollierbar sind. Beide Dimensionen müssen zusammentreffen, sind aber unterschiedlich voraussetzungsstark. Die gesellschaftliche und wirtschaftliche Wirksamkeit ergibt sich aus dem Grad existenzieller Bedeutung für andere Bereiche respektive dem Grad existenzieller Bedrohung durch den Lockdown. Die Bedeutung hängt dabei wesentlich von der Betrachtung der Vorleistungs-, Produktions- und Vertriebsketten ab.

Die epidemiologische Beherrschbarkeit und Kontrollierbarkeit bezieht sich neben der allgemeinen wie regionalen Entwicklung der Fallzahlen auf die Infrastruktur für das Testen und Tracken. Hier geht es z. B. auch um die ablauforganisatorischen Anpassungsmöglichkeiten an gebotene Abstandsregeln oder Schutzvorrichtungen. Damit erhalten Schul- und Gewerbeaufsicht, Arbeitsschutz etc. neue Verantwortlichkeiten zur Sicherstellung der Vorsorgestandards.

Beachtenswert ist dabei auch, dass die verschiedenen Wirtschaftszweige einem unterschiedlichen Druck ausgesetzt sind und einen zeitlich divergierenden Handlungsbedarf für die Normalisierung und die wirtschaftspolitische Unterstützung begründen (vgl. Tabelle 2). Während Landwirtschaft, Handel und Dienstleistungen zwar einen hohen Druck verspüren, aus dem Lockdown auszusteigen, weil es um fundamentale, existenzielle Fragen des Fortbestands von Unternehmen und Arbeitsplätzen geht, so bleiben die weiteren wirtschaftspolitischen Maßnahmen überschaubar. Kurzarbeitergeld, Steuerstundungen, Kredite mit weitgehender Haftungsübernahme dürften in der Öffnungsphase hinreichend sein und fortlaufend an Bedeutung verlieren. Damit gewinnt der Staat Freiraum für die gezielte Förderung und Unterstützung längerfristig in schwieriger Lage befindlicher Wirtschaftszweige.

Dazu gehört sicher die Industrie, die kurzfristig wegen ihrer besseren Eigenmittelausstattung, der leichteren Nutzbarkeit der Kurzarbeitergeldregelung und der Möglichkeit, wenigstens partiell aus unterschiedlicher regionaler oder sektorspezifischer Entwicklung Abfederungen zu

Tabelle 2

**Restriktionen und Re-Start-Potenziale der Sektoren**

| | | Angebot | Nachfrage | Zeitpfad |
|---|---|---|---|---|
| | Landwirtschaft | Verfügbarkeit von Saisonkräften | Eventuell Rückgang durch Handelsbeschränkungen | Kurzfristig, wenn Saisonkräfte verfügbar |
| | Verarbeitendes Gewerbe | Störungen in internationalen Wertschöpfungsnetzen, Handelsbeschränkungen, Verfügbarkeit der Mitarbeiter (Homeoffice, Gesundheitsschutz) | Rückgang bei nachlassender Weltwirtschaft, Störungen bei Abnehmern, Zurückhaltung bei Investitionen und beim Kauf langlebiger Konsumgüter | Längerfristige Störung, schwieriger Rückgang zur Normalsituation |
| | Bauwirtschaft | Verfügbarkeit von Subunternehmern aus der EU, ggf. Lieferung von Material | Zurückhaltung bei Investitionen und privater Modernisierung | Längerfristig vor allem auf der Nachfrageseite |
| | Energieversorgung | Bisher geringe Angebotsprobleme | Deutliche Nachfragerückgänge | Schnelle Reaktion bei steigendem Bedarf möglich |
| Dienstleistungen | digitalisierbar | Kapazitätsgrenzen bei Netzen; Verlagerung von analogen zu digitalen Angeboten | Hohe Nachfrage durch Digitalisierungsschub | Kurzfristig und nachhaltig |
| | Sozialkonsum | Restriktionen zur Krankheitsbekämpfung (Abstand, Veranstaltungsgröße) | Im Wesentlichen schnell reaktivierbar | Kurz- bis langfristig, je nach Auflagen |
| | personenbezogen | Verbote zur Krankheitsbekämpfung | Schnell reaktivierbar | Kurz- bis langfristig, je nach Auflagen |

Quelle: eigene Zusammenstellung.

organisieren, robuster sein dürfte, mittelfristig jedoch wegen drohender Asynchronität der internationalen Märkte sowie der Abhängigkeit der Lieferketten einer stärkeren Unterstützung bedarf. Derzeit wird in weiten Bereichen (fern der Automobilbranche) des Verarbeitenden Gewerbes noch produziert – das gilt für einen Teil der Chemischen Industrie, die Elektrotechnik und den Maschinenbau. In der Industrie erweist sich die Globalisierung/Europäisierung der Wertschöpfungsketten nun als besondere Herausforderung.

Als volkswirtschaftliche Schlüsselbranche in der Krise erweist sich die Automobilindustrie. Hier ruht die Produktion seit Mitte März, da es umfassende Probleme sowohl auf der Beschaffungsseite (Vorleistungen, Ausfall Beschäftigung) als auch auf der Absatzseite (Händlernetz, Kfz-Zulassungsstellen, Kaufzurückhaltung bei langlebigen Konsumgütern) gibt. Der Umsatz der Branche lag 2019 bei rund 436 Mrd. Euro (283 Mrd. Euro im Ausland, 153 Mrd. Euro im Inland; Exportquote 65 %; 82 Mrd. Euro war der Umsatz der Zulieferer); die Zahl der Beschäftigten betrug 2019 833.000 (Zulieferer über 300.000). 27 % der weltweit in der Branche angemeldeten Patente fallen auf die deutsche Autoindustrie. Zugleich zeichnen die Autohersteller für 40 % aller Patentanmeldungen aus Deutschland verantwortlich und stellen damit die mit Abstand innovativste aller Branchen.

Daraus ergeben sich Hinweise für die gesellschaftliche und wirtschaftliche Bedeutung, die bei einer schrittweisen Öffnung zu berücksichtigen und mit der epidemiologischen Beherrschbarkeit zu verbinden sind. Deshalb steht am Beginn der Öffnungsphase die Sicherstellung der weithin akzeptierten medizinischen Infrastrukturen.

## Mögliche Maßnahmen in der Öffnungsphase

Alle Maßnahmenplanungen können nur vorläufiger Natur sein. Eine Abschätzung der Folgen eines wirtschaftlichen Einbruchs für verschiedene Zielgruppen sowie der Wirksamkeit der Gegenmaßnahmen sollte so früh wie möglich auf den Weg gebracht werden. Klar ist, dass jeder Schritt viel Innovationskraft erfordert, um Stück für Stück eine Öffnung hinzubekommen, ohne Ansteckungsrisiken unangemessen zu vergrößern. Aus aktueller Perspektive erscheinen folgende zwölf Punkte besonders bedeutsam:

### 1. Tests und medizinische Behandlungskapazitäten aufbauen, Schutzkleidung vorhalten

Die großflächige Erhöhung von Test- und Behandlungskapazitäten (Test-Kits und Laborkapazitäten, Tracing-Software, medizinischer Sauerstoff, Beatmungsgeräte, Intensivbetten, Personal) ermöglicht einen schnelleren Exit aus dem Lockdown und ist daher ein notwendiger erster Schritt zur wirtschaftlichen Normalisierung. *Gesellschaftlich-ökonomische Wirksamkeit:* groß, weil zentrale Voraussetzung für alle Folgeschritte; *epidemiologische Beherrschbarkeit:* unbestritten notwendig.

### 2. Öffentliche Verwaltung öffnen

Die öffentliche Verwaltung ist zügig wieder zu öffnen, auch für den Besuchsverkehr. Dies normalisiert das öffentliche Leben, hat aber auch im Fall von Kfz-Zulassungsstellen direkte wirtschaftliche Relevanz. Schutzmaßnahmen, wie sie in Supermärkten oder Apotheken getroffen wurden, können auch hier die Ansteckungsgefahr verringern. *Gesellschaftlich-ökonomische Wirksamkeit:* groß, weil damit ein Signal an die Privaten gegeben wird, dass die notwendige Unterstützung und Begleitung gegeben sind; *epidemiologische Beherrschbarkeit:* Testkapazitäten für Publikumsbereiche; ablauforganisatorische Begleitung.

### 3. Schulen und Kindergärten öffnen

Für eine möglichst schnelle Öffnung müssen Wege jenseits der bisher gekannten Organisationsformen gefunden werden. Der Verzicht von Präsenzunterricht für die Abiturienten schafft zusätzliche Kapazitäten, durch Unterricht im Schichtbetrieb am Vor- und Nachmittag kann die Schülerzahl reduziert werden, die gleichzeitig vor Ort ist. Sicherzustellen ist, dass Lehrer aus Risikogruppen (Alter, Vorerkrankung) vom Unterricht freigestellt werden. *Gesellschaftlich-ökonomische Wirksamkeit:* von fundamentaler ökonomischer Bedeutung, weil Kinderbetreuung oder gar Homeschooling parallel zum Homeoffice keine adäquaten Alternativen sind, und die Bildungsgerechtigkeit abnimmt, weil die Leistungsunterschiede zwischen den Kindern in Abhängigkeit zum Elternhaus weiter zunehmen; *epidemiologische Beherrschbarkeit:* Ablauforganisatorische Voraussetzung wichtig; ein flächendeckender Schnelltest für alle Schüler kann als Bedingung für die Öffnung der Schule vorgesehen werden.

### 4. Verkehrskapazitäten erhöhen

Die heruntergefahrene Verkehrsinfrastruktur muss schnell erhöht werden, um den Weg zur Arbeit zu ermöglichen. Eine höhere Taktung reduziert die Zahl der Menschen im jeweiligen Bus oder der Bahn. Durch improvisierte Abtrennungen können möglicherweise Abteile geschaffen werden, wodurch sich die soziale Distanz zwischen den kleineren Gruppen von Reisenden erhöhen lässt. *Gesellschaftlich-ökonomische Wirksamkeit:* groß, weil infrastrukturelle Voraussetzung für alle anderen Schritte; *epidemiologische Beherrschbarkeit:* Schutz der Mitarbeiter der Betriebe (Testing), organisatorische Vorkehrungen.

### 5. Einzelhandel öffnen und Dienstleistung zulassen

Sobald wie möglich sollen Handelsgeschäfte und derzeit untersagte Dienstleistungen wieder zugelassen werden. Die unterschiedliche Vorgehensweise in den Bundesländern spricht dafür, dass der Handel nicht so weitgehend geschlossen sein muss, wie dies teilweise der Fall ist. Für die Bereiche, bei denen von längerfristigen Beschränkungen oder Verboten auszugehen ist, ist Klarheit über die Perspektiven erforderlich. Zudem muss über massive Unterstützungsleistungen nachgedacht werden. Sollten Großveranstaltungen noch über viele Monate untersagt bleiben, würde dies sonst zu einer Konkurswelle unter Messebauern, Catering-Unternehmen, Event-Veranstaltern etc. führen. *Gesellschaftlich-ökonomische Wirksamkeit:* groß, weil der öffentliche Raum seine integrierende, steuernde und kommunikative Wirkung wieder entfalten kann; *epidemiologische Beherrschbarkeit:* Abstands- und Zugangsregeln beibehalten. In den Sommermonaten sollte großzügig mit Servicemöglichkeiten auf Bürgersteigen und Plätzen umgegangen werden.

### 6. Produktion in der Industrie hochfahren

Um die industrielle Produktion wieder hochfahren zu können, müssen komplexe Wertschöpfungsnetze reaktiviert werden. Dazu ist erforderlich, dass die Unternehmen trotz der krisenbedingten Liquiditätsschwierigkeiten weiterhin am Markt sind. Teilweise müssen zudem Vertriebskanäle wieder zugelassen werden und öffentliche Zulassungsstellen wieder arbeiten. Um die Wertschöpfungsnetzwerke möglichst friktionsfrei wieder anfahren zu können, bedarf es zudem eines klaren Signals, wann wieder mit einer Normalisierung gerechnet werden kann. Darauf aufbauend können die notwendigen Koordinierungen vorgenommen werden. Dies sollte soweit möglich im europäischen Verbund geschehen, da die Produktion in Europa stark länderübergreifend vernetzt ist. *Gesellschaftlich-ökonomische Wirksamkeit:* groß, weil so die bedeutsamen Wertschöpfungssektoren wieder anlaufen, Kurzarbeit nicht zu Arbeitslosigkeit wird. Es sind auch Ausstrahleffekte auf den internationalen Handel zu erwarten, weil Vorleistungen nachgefragt werden. Steuerbasis wird zumindest teilweise gesichert; *epidemiologische Beherrschbarkeit:* Notwendig ist, dass die Mitarbeiter in Produktion, Logistik, Sales und After-Sales sicher und ansteckungsfrei ihrer Arbeit nachgehen können. Arbeitsorganisatorische Anstrengungen in Betrieben sind notwendig, um neue Standards für Gesundheitsschutz schnell zu erfüllen.

### 7. Offene Grenzen sichern

Die komplexen Wertschöpfungsketten basieren auf internationaler Arbeitsteilung und damit verbunden dem internationalen Austausch von Gütern. Grenzen für Waren müssen offengehalten und wo notwendig wieder friktionsfrei geöffnet werden, das gilt auch für den grenznahen Arbeitskräfteaustausch. Hier liegt eine wichtige Aufgabe für die Europäische Union. Internationaler Austausch über den Luft-, See- und Landweg muss gewährleistet werden. *Gesellschaftlich-ökonomische Wirksamkeit:* groß, weil damit die europäische Koordinierung gestärkt wird und Asynchronitäten eingedämmt werden; *epidemiologische Beherrschbarkeit:* Priorität für den Warenhandel, Testing für grenzüberschreitende Arbeitskräftemobilität.

## Perspektiven auf die Stabilisierungsphase

Die Öffnungsphase ist auch eine Versuchsphase und ermöglicht das Lernen sowie Adjustieren der politischen Regulierungen. Danach wird sich der Blick schnell auf die Frage richten, ob und wie der Weg zu einer neuen Normalität gelingt. Es ist völlig klar, dass es nach dieser Krise keine Rückkehr zur alten Normalität gibt. Auf dem Weg zum „New Normal" verbinden sich die Anpassungsleistungen der Unternehmen mit dem Bedarf an Innovationen und neuen Geschäftsmodellen, die sich mitunter auch ohne die Krise aufgedrängt hätten. Gleichzeitig wird es Branchen geben, in denen aus Gründen des Infektionsschutzes noch länger mit fundamentalen Restriktionen zu rechnen ist oder die sich in einer Anpassungskrise hin zu einem deutlich niedrigeren Marktvolumen befinden werden. Dies kann längere wirtschaftspolitische Absicherungs- oder Anpassungsmaßnahmen erfordern. Zudem steht die Wirtschaftspolitik vor einer weitergehenden Herausforderung: Denn es geht nicht mehr um kurzfristige Absicherung, Stabilisierung und Stützung, sondern nun um die Stärkung der gesamtwirtschaftlichen Dynamik und die Schaffung von Voraussetzungen für zukunftsfähige Wertschöpfung am Standort Deutschland. In der Stabilisierungsphase zum „New Normal" sind die folgenden Maßnahmen und Schritte bedeutsam. Diese bereits jetzt mitzudenken, ist aus zwei Gründen geboten: Einerseits stabilisiert dies die Erwartungen der privaten Akteure und schafft Vertrauen, andererseits muss der Staat bei seinen wirtschafts- und finanzpolitischen Interventionen einen langen Atem beweisen. Nur mit Blick auf die künftigen Herausforderungen wird das gelingen.

### 8. Großzügig mit fiskalischen Forderungen umgehen

Nach dem Wiederanfahren wird in vielen Unternehmen die Liquiditätssituation weiterhin kritisch sein, zumal wenn verlorene Umsätze nicht nachgeholt werden können. Um die Unternehmen nicht zu gefährden, muss die öffentliche Hand sich bei Steuernachzahlungen, Sozialversicherungsbeiträgen und Rückzahlungen von Überbrückungskrediten großzügig verhalten. Gegebenenfalls ist ein Forderungsverzicht bei Fortführung der Geschäftstätigkeit zu prüfen. Eine Lösung für den Schuldenüberhang der Unternehmen ist zu prüfen. *Gesellschaftlich-ökonomische Wirksamkeit:* wichtig wegen der Nachhaltigkeit der Förderung; *epidemiologische Beherrschbarkeit:* unbedenklich.

### 9. Einreise ausländischer Arbeitnehmer möglich machen

Verschiedene Branchen sind auf ausländische Arbeitskräfte oder Subunternehmer angewiesen. Dies gilt beispielsweise für die Landwirtschaft und die Bauwirtschaft. *Gesellschaftlich-ökonomische Wirksamkeit:* groß wegen der Sicherung der Wertschöpfung in zentralen Wirtschaftszweigen; *epidemiologische Beherrschbarkeit:* Vorrangregelungen und Schnelltests für diese Gruppen können dazu beitragen, eine sichere Einreise rechtzeitig zu ermöglichen.

### 10. Fiskalischen Startschuss geben und Nachfrageprogramm vorbereiten

Durch eine steuerliche Entlastung kann ein Aufbruchssignal an Konsumenten und Unternehmen gesendet werden. Die vollständige Abschaffung des Solidaritätszuschlags zum Sommer 2020 wäre ein solches Signal, das wie ein Startschuss für einen neuen Aufschwung wirken kann. Darüber hinaus sind fiskalische Impulse im Sinne der Konjunkturpolitik zu prüfen, um aus der Öffnungsphase einen robusten Pfad zum „New Normal“ zu ermöglichen. Aufgrund der negativen Erwartungsbildung in der Bevölkerung kann es zu einer Situation kommen, in der langlebige Konsumgüter nicht nachgefragt oder Investitionen nicht getätigt werden. Wenn sich dies auch nach Aufheben der Angebotsrestriktionen als krisenverlängerndes Problem erweist, kann dem mit spezifischen Programmen zur Nachfragestimulation begegnet werden, wie dies auch in der Wirtschaftskrise 2009 erfolgreich war. *Gesellschaftlich-ökonomische Wirksamkeit:* groß wegen der Sicherung der Wertschöpfung in zentralen Wirtschaftszweigen; *epidemiologische Beherrschbarkeit:* unbedenklich.

## Perspektiven auf die Wachstumsphase

In der mittel- bis langfristig ausgelegten Wachstumsphase sind Instrumente der Wachstums- und Innovationspolitik verstärkt zu aktivieren.

### 11. Wachstumsprogramm aufsetzen

Die Corona-Krise ist vermutlich auf Jahre ein spürbarer Rückschritt in der Wohlstandsentwicklung Deutschlands. Dies trifft die deutsche Volkswirtschaft in einer Zeit, in der sie ohnehin vor einem großen Strukturwandel und disruptiven Veränderungen steht. Damit wird ein umfangreiches Programm zur Verbesserung der Standortbedingungen und zur Stärkung der Disruptionsfähigkeit notwendig, um durch angebotsseitige Maßnahmen Investitionen, Innovationen und zukünftiges Wachstum zu ermöglichen. Der Staat beteiligt sich – auf die Anfangsphase begrenzt – an den risikoträchtigen Innovationen im Bereich Gesundheit, Energie, Klima und setzt damit Signale. *Gesellschaftlich-ökonomische Wirksamkeit:* groß wegen der Sicherung der Wertschöpfung in zentralen Wirtschaftszweigen; *epidemiologische Beherrschbarkeit:* unbedenklich.

### 12. Handlungsfähigkeit des Staates sichern

Die fiskalische Beanspruchung des Staates durch die Krise wird mit einem erheblichen Anstieg der öffentlichen Schulden einhergehen. Die Schuldentragfähigkeit der öffentlichen Haushalte wird zu einem wichtigen Argument der Zukunftssicherung. Deshalb sind ab einem bestimmten Zeitpunkt Schritte zur Sanierung der öffentlichen Haushalte einzuleiten. Dazu gehört auch eine neue Würdigung des Problems der kommunalen Schulden, die durch die Krise sicherlich kräftig ansteigen werden. *Gesellschaftlich-ökonomische Wirksamkeit:* groß wegen der positiven Erwartungsstabilisierung der Privaten; *epidemiologische Beherrschbarkeit:* unbedenklich.

**Title:** ***An Economic Policy Exit Strategy from the Corona Lockdown***

**JEL Classification:** *O40, I15, H12*
